# Future of ADHD Care: Evaluating the Efficacy of ChatGPT in Therapy Enhancement

**DOI:** 10.3390/healthcare12060683

**Published:** 2024-03-19

**Authors:** Santiago Berrezueta-Guzman, Mohanad Kandil, María-Luisa Martín-Ruiz, Iván Pau de la Cruz, Stephan Krusche

**Affiliations:** 1 Applied Software Engineering Research Group, School of Computation, Information, and Technology, Technical University of Munich, 80333 Munich, Germany; mohanad.kandil@tum.de (M.K.); krusche@tum.de (S.K.); 2 Grupo de Investigación Innovación Tecnológica para las Personas (InnoTep), Departamento de Ingeniería Telemática y Electrónica, ETSIS de Telecomunicación, Campus Sur, Universidad Politécnica de Madrid, 28031 Madrid, Spain; marialuisa.martinr@upm.es (M.-L.M.-R.); ivan.pau@upm.es (I.P.d.l.C.)

**Keywords:** artificial intelligence, LLMs, cognitive therapy, ADHD, ChatGPT, customizable AI bots, robotic systems in therapy, sensory data integration, AI-driven decision making, occupationaltherapy innovation, personalized therapy sessions, AI in mental health, computational cognitive tools

## Abstract

This study explores the integration of large language models (LLMs), like ChatGPT, to improve attention deficit hyperactivity disorder (ADHD) treatments. Utilizing the Delphi method for its systematic forecasting capabilities, we gathered a panel of child ADHD therapy experts. These experts interacted with our custom ChatGPT through a specialized interface, thus engaging in simulated therapy scenarios with behavioral prompts and commands. Using empirical tests and expert feedback, we aimed to rigorously evaluate ChatGPT’s effectiveness in therapy settings to integrate AI into healthcare responsibly. We sought to ensure that AI contributes positively and ethically to therapy and patient care, thus filling a gap in ADHD treatment methods. Findings show ChatGPT’s empathy, adaptability, and communication strengths, thereby highlighting its potential to significantly improve ADHD care. The study points to ChatGPT’s capacity to transform therapy practices through personalized and responsive patient care. However, it also notes the need for enhancements in privacy, cultural sensitivity, and interpreting nonverbal cues for ChatGPT’s effective healthcare integration. Our research advocates for merging technological innovation with a comprehensive understanding of patient needs and ethical considerations, thereby aiming to pioneer a new era of AI-assisted therapy. We emphasize the ongoing refinement of AI tools like ChatGPT to meet ADHD therapy and patient care requirements more effectively.

## 1. Introduction

Attention deficit/hyperactivity disorder (ADHD) is a prevalent neurodevelopmental disorder that poses substantial challenges to individuals’ daily functioning [1]. Recent advances in therapeutic interventions have broadened the scope of treatment beyond conventional pharmacological approaches, thereby integrating novel and innovative methods [2,3].

Among these emerging therapies, *music therapy* has demonstrated that it can improve overall well-being in individuals with ADHD [4], cognitive behavioral therapy (CBT) has been recognized as a practical approach in reducing ADHD symptoms in adults [5], and robotic assistance shows potential in providing effective and nonpharmacological interventions for individuals with ADHD, thereby offering promising avenues for enhancing the quality of life and functional outcomes of affected individuals [6].

This research explores the integration of large language models (LLMs), such as OpenAI’s ChatGPT, to enhance and supplement nonpharmacological therapies for children with ADHD. It outlines the essential criteria for developing tailored tools catering to the distinct requirements of individuals with ADHD. Our study aims to forge more pervasive, interactive, and personalized therapeutic solutions to enhance engagement and overall effectiveness. Considering the limitations posed by traditional therapy, which include high costs, location constraints, and lengthy wait times, ChatGPT would serve as a transformative tool that overcomes all these limitations. It would offer immediate, round-the-clock support, which is particularly valuable for individuals in remote or underserved areas. Integrating ChatGPT into therapeutic practices would broaden access to mental health support, thus ensuring that more individuals could benefit from tailored and engaging therapy experiences.

This research examines the theory, practical use, and ethical aspects of combining LLMs (such as ChatGPT) and robotic assistance to significantly contribute to ADHD nonpharmacological therapies in intelligent environments. Therefore, the Section 1.1 and Section 1.2 provide a detailed review of LLMs supporting cognitive therapies and human–machine interfaces in robotic assistants. In Section 1.3, we explain our previous work using robotic assistants for supporting ADHD therapies and the limitations that ChatGPT will help to overcome, such as enhancing the interactivity of therapy sessions. Section 2 explains how ChatGPT has been integrated to support ADHD therapies and is being integrated into robotic assistants. Section 2.2 explains the therapeutic validation by experts to assess the feasibility of ChatGPT in an ADHD therapy process using the *Delphi method*. The results are presented in Section 3 and discussed in Section 4, where we outline the potential future directions and challenges for research in this area. Finally, the conclusions are presented in Section 5.

### 1.1. LLMs Supporting Cognitive Therapies

Recent advancements in AI and natural language processing (NLP) have opened new avenues in the field of cognitive therapies, particularly for individuals with cognitive disabilities [7]. Among these advancements, LLMs like ChatGPT have emerged as potential tools for supporting cognitive processes and enhancing learning experiences [8,9].

A notable study by Tamdjidi and Billai at the KTH Royal Institute of Technology investigated this aspect, where they specifically focused on using ChatGPT as an assistive technology in reading comprehension for individuals with ADHD. Participants with and without ADHD were assessed through reading comprehension tests conducted with and without the assistance of ChatGPT. This study provided insights into the effectiveness of ChatGPT in aiding reading comprehension. The intriguing findings revealed a general decrease in comprehension abilities when ChatGPT was used as a learning aid across both groups. However, an interesting pattern emerged among participants with prior ChatGPT experience. Indeed, these individuals exhibited an increase in their comprehension abilities. This suggests that familiarity and experience with the tool significantly affect its effectiveness as an assistive technology. Overall, the research presents a nuanced view of the potential and limitations of ChatGPT in cognitive therapies, particularly in enhancing reading comprehension for individuals with ADHD [10].

According to the research by Cho and Kim, LLMs effectively engaged patients empathetically and adaptively. However, it also identified limitations in personal interactions and understanding emotions to the depth that a human therapist would do. Overall, the study highlighted the potential of LLMs as supportive tools in therapy while emphasizing the need for further development to enhance their capabilities in building deeper therapeutic relationships and tailoring responses to individual needs based on their feelings [11].

The study developed by Lin et al. presented *Healthy AI* as a safety framework for integrating LLMs into applications. This approach defines *Healthy AI* as integrating safety, trustworthiness, and ethical alignment with human values. Indeed, the focus is on creating healthy AI systems that perform efficiently and adhere to social norms and ethical standards. The development of LLMs emphasizes the need for AI systems to be transparent and accountable, thus prioritizing the well-being of users while aligning with societal values and expectations. Such an approach is crucial for fostering trust and reliability in LLMs, thereby making them more beneficial and acceptable in the various applications where they are used, from customer service to mental health support and cognitive therapies [12].

The research conducted by Gabor-Siatkowska et al. explored the innovative application of ChatGPT to enhance the training of *Terabot*, a dialogue system designed for therapeutic interactions with psychiatric patients. The core of the study lies in leveraging ChatGPT to generate additional training data, which subsequently led to a significant improvement in the system’s ability to recognize user intents. Marked by a 13% increase in accuracy, this advancement underscores the potential of utilizing AI models like ChatGPT not only as tools for direct interaction but also as resources for augmenting the capabilities of other AI-driven systems, particularly in sensitive areas such as mental health therapy [13].

In the same field, Moraiti and Drigas discussed the potential of AI tools, specifically ChatGPT, to assist individuals with neurodevelopmental disorders. They highlighted the benefits of such technologies in providing personalized learning experiences, assessments, and diagnoses. While these AI tools show promise in enhancing support and interventions for people with neurodevelopmental disorders, the authors underscore that they are not replacements for human therapists and healthcare professionals. The study suggests that AI can complement traditional therapeutic methods, thus potentially improving the accuracy and effectiveness of treatments for this population [14].

In the study by Kim et al., the efficacy of ChatGPT 3.5 in diagnosing and recommending treatments for developmental and behavioral pediatrics (DBP) was evaluated through its performance on 97 DBP case studies. A panel of three DBP physicians reviewed ChatGPT’s outputs, thus assessing diagnostic accuracy, treatment recommendation accuracy (5-point Likert scale), completeness (3-point Likert scale), and the ChatGPT’s consideration of cultural and ethical issues. The results highlighted ChatGPT’s strengths in formulating detailed treatment plans (4.6/5). Still, they noted a significant shortfall in diagnostic accuracy (66.2% of the case reports), thereby emphasizing the irreplaceable value of professional medical consultations [15].

It is essential to keep checking and improving AI models to make sure they can create medical information that is trustworthy and safe, primarily when used for mental health therapies and other medical uses. Doing this is critical to making people trust these models and finding them helpful in different medical areas [16].

### 1.2. LLMs Meet Robotics to Support Cognitive Treatments

Since the rise of LLMs, numerous adjacent fields have recognized the opportunity to enhance their scope with their implementation. Robotics is not an exception [17].

The integration of ChatGPT into robots has significantly increased trust in human–robot collaboration in several scenarios. This improvement is attributed to the robot’s enhanced ability to communicate more effectively with humans thanks to ChatGPT’s proficiency in understanding the nuances of human language and responding appropriately [18].

The study by Sai Vemprala et al. presented an experimental study focusing on the integration of ChatGPT in robotics. It delves into various prompt engineering techniques and dialogue strategies to assess ChatGPT’s capability to effectively execute a wide range of robotics tasks. Through *prompt* engineering and strategic dialogue management, ChatGPT demonstrates significant promise in interpreting and executing complex commands, thereby expanding the scope of tasks that robots can perform. This breakthrough has implications for the future of human–robot interaction, thus potentially making robots more accessible and easier to control through natural language commands. This research highlighted the potential of leveraging advanced conversational AI models like ChatGPT to enhance the functionality and versatility of robotic systems [19].

The study conducted by Bertacchini et al. explored the application of social robotics (using a Pepper robot) connected to the ChatGPT for real-time dialogue initiation with individuals with autism spectrum disorder (ASD). This study is part of a broader research landscape focusing on the potential benefits of integrating social robots in therapy and educational programs for individuals with ASD. The study underlines the potential benefits of such integrated systems for ASD interventions. It also emphasizes the need for further research to assess their effectiveness, feasibility, and acceptability in real-world settings [20].

In the same field, the study by Viviane Kostrubiec et al. found that children with ASD exhibited more progress in their theory of mind skills after interacting with a human partner compared to a social robot, thus indicating the nuanced dynamics between human–robot and human–human interactions in therapeutic contexts [21]. Another study highlighted using a social robot to teach music to children with low-functioning autism, thereby decreasing stereotyped behaviors and improving various social and cognitive skills [22]. These examples underscore the potential and challenges of leveraging technology, including social robots connected with advanced conversational AI like ChatGPT, to support the development and well-being of individuals with ASD.

Exploring the legal practices regarding control and security in using ChatGPT for cognitive therapies highlights the broader concerns surrounding generative artificial intelligence (GAI) technologies. A critical analysis by K. Wach et al. on the dark side of generative AI, including technologies like ChatGPT, sheds light on several controversies and threats, such as the lack of regulation, issues of poor quality control, the risk of job losses due to automation, violations of personal data, potential for social manipulation, increasing socioeconomic inequalities, and AI-induced technostress. These findings underscore the pressing need for regulatory frameworks to ensure fair competition, protect privacy and intellectual property rights, and mitigate potential risks associated with deploying GAI technologies in sensitive areas such as cognitive therapies [23].

The absence of studies directly investigating the integration of LLMs like ChatGPT within ADHD therapy underscores a critical gap in current research. Despite the extensive exploration of LLMs across various sectors—including their deployment in enhancing performance, their emergent abilities, and their implications for education and healthcare—the specific application of these technologies for diagnosing, supporting, and intervening in ADHD remains largely unexplored. This oversight signals a pressing need to delve into how ChatGPT and similar AI tools could be tailored to meet the unique needs of individuals with ADHD, especially considering their potential in child and adolescent mental health contexts.

LLMs’ versatility and advanced capabilities in reasoning, language understanding, and content generation highlight their potential utility in mental health interventions. Yet, the literature’s silence on their direct application to ADHD therapy points to an urgent need for focused research. This uncharted territory holds promise for groundbreaking advancements in mental health care, thus inviting a thorough investigation into how ChatGPT and other AI models can be harnessed to support ADHD treatment and understanding and filling a significant gap in the scientific literature and in clinical practice.

### 1.3. Preliminary Developed Work

Robotics has had a significant impact on supporting individuals with ADHD, particularly with activity treatments [24,25,26], language development [27], therapeutic purposes [28,29,30,31,32], learning outcomes [33,34], and the development of attention and memory [35].

In previous research, we have developed a robotic assistant that accompanies children with ADHD while completing their school tasks at home (homework). This robotic assistant, along with motion and proximity sensors located on the desk and chair, can determine distraction events exhibited by the child, such as playing with the chair or moving away from the workspace [36,37]. It also identifies smaller-scale distraction events, like playing over the desk or daydreaming [38]. The collected information about the kids’ behavior and performance during the homework activities was processed and presented in charters to therapies and parents through a mobile application to further analyze and evaluate the treatment [39].

Depending on the frequency of these distraction events, the robotic assistant provides feedback to the child to regain their attention on the task at hand [40]. One significant limitation identified in the feasibility analysis of the robotic assistant was its tendency to become predictable and, consequently, less engaging over time, as highlighted in [34]. This issue stemmed primarily from the robot’s reliance on a finite set of prerecorded dialogues and instructions. As a result, the interactions offered by the robot inevitably turned repetitive after prolonged use. This repetitiveness not only diminished the robot’s effectiveness in maintaining the interest and attention of children with ADHD but also limited its potential as a dynamic and adaptive tool in therapeutic settings. Addressing this drawback is crucial for enhancing the robot’s long-term utility and effectiveness in engaging with its young users.

The advent of generative AI and LLMs has revolutionized cognitive therapies (as detailed in Section 1.1) and human–machine interaction (outlined in Section 1.2). Therefore, we explored the potential to integrate ChatGPT into the human–machine interaction component of our robotic assistant to address and overcome the challenges previously identified in the field, as documented in [34]. Incorporating ChatGPT into our robotic assistant would enhance its interactive capabilities and promise to refine the therapeutic process, thus offering a more engaging and practical experience for children undergoing therapy. Therefore, we created a *custom GPT* for our purpose.

### 1.4. Custom GPTs

The research by Konstantin Hebenstreit et al. explored the effectiveness of chain-of-thought (CoT) reasoning in LLMs [41], particularly in zero-shot learning scenarios. Key findings revealed that specific prompts like *“Let’s think step by step”* significantly enhance the models’ reasoning and problem-solving abilities across various models and datasets. Moreover, this approach is especially beneficial for the GPT-4 model in outperforming direct prompting methods. It also emphasized the need for more refined datasets to challenge these models adequately. Overall, the research demonstrated the crucial role of well-structured prompts in boosting the performance and obtaining the efficient output of LLMs in complex question-answering tasks. Moreover, to build a conservative model with efficient outputs, the model must adapt the power of verbal reinforcement learning.

**Figure 1:**
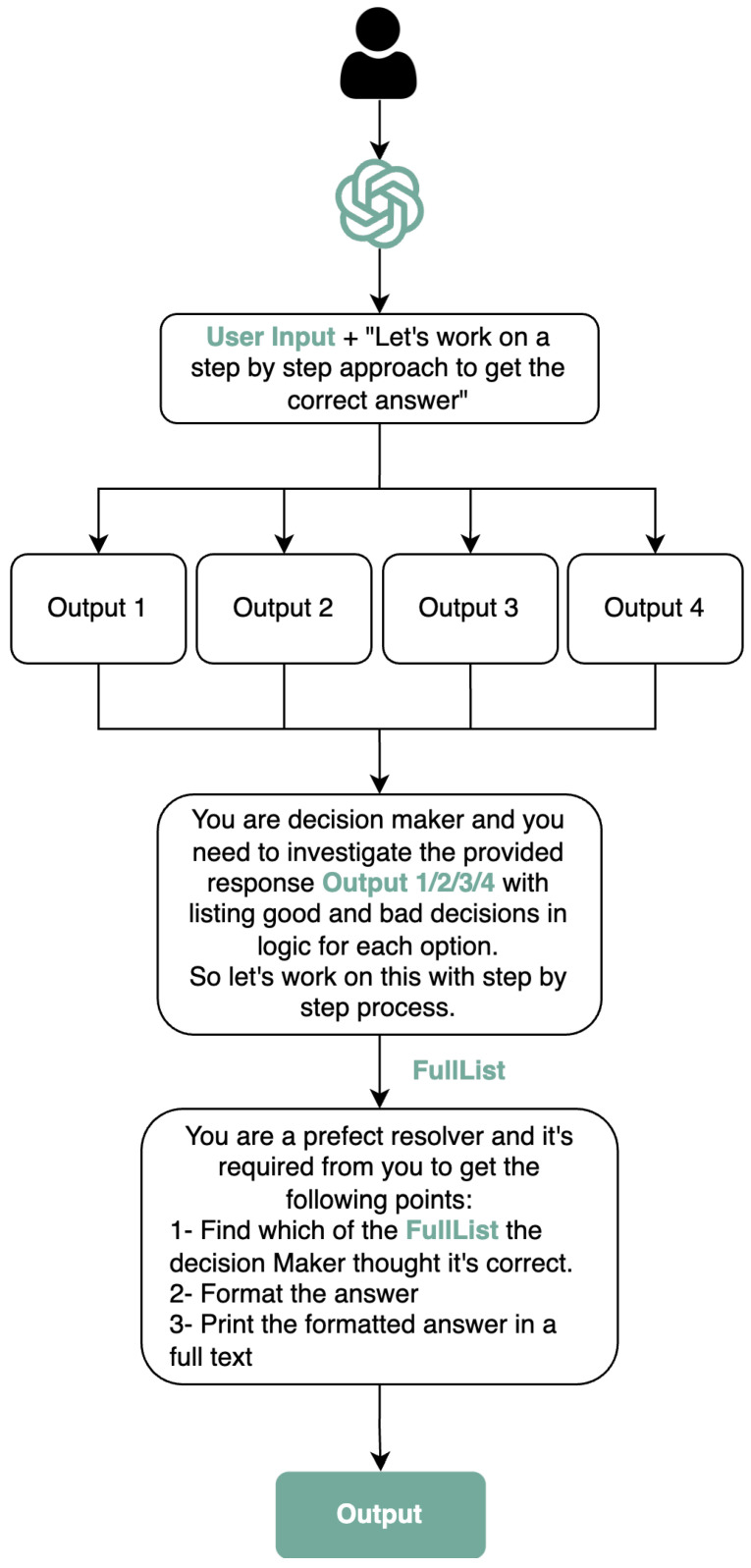
Workflow for customizing ChatGPT: User inputs prompts AI to produce options. A decision maker evaluates these, and a resolver finalizes the best response.

Figure 1 illustrates an AI-driven decision-making process where the user inputs an AI model, which prompts the system to “Let’s work on a step-by-step approach to get the correct answer”. The AI model then generates multiple outputs for evaluation. As a decision maker, the model investigates these outputs, thus assessing the logic behind each to identify the advantageous and disadvantageous decisions embedded within them. This reflective evaluation is aimed at a step-by-step refinement to reach the optimal decision. Following this, the customGPT, acting as a resolver, is tasked with distilling the insights from the FullList—an aggregation of the AI-generated outputs deemed correct by the decision-making process. The resolver’s objectives are to format the chosen answer correctly and present it as a fully articulated text, thereby transforming the AI-assisted decisions into a coherent and finalized response.

The study by Shinn et al. presented *Reflexion*, a groundbreaking framework that enhances the learning efficiency of LLMs in interactive environments, such as games and software APIs (https://github.com/noahshinn/reflexion, accessed on 21 January 2024) (application programming interfaces). Unlike traditional reinforcement learning, which demands extensive training samples and fine-tuning, *Reflexion* leverages linguistic feedback in the model. This method involves language agents reflecting on their performance, storing these reflections in memory, and using this accumulated knowledge to improve decision making in subsequent attempts [42].

Remarkably, *Reflexion* showed substantial improvements in diverse tasks like sequential decision making, coding, and language reasoning. This last improvemeny would be beneficial for the creation of a novel therapy interaction approach for children with ADHD due to *Reflexion* achieving a 91% accuracy on the HumanEval coding benchmark, thus surpassing GPT-4’s 80%. The framework is adaptable to various feedback types and sources, thus making it a versatile tool for reinforcing language agents. This advancement opens new pathways for efficient, context-aware learning in AI systems, thereby showcasing a significant leap in artificial intelligence.

Finally, simple procedures in the model’s internal system can produce an output with zero-shot learning, as illustrated in Figure 1. This approach can maximize the likelihood of getting correct reasoning steps and generating an efficient output by zero-shot prompts. Indeed, this approach is included in our upcoming testing phases to obtain perfect reasoning with filtered results for ADHD patients.

## 2. Materials and Methods

Based on the literature review, we developed a custom ChatGPT to be validated by therapeutic experts before being implemented in a robotic assistant to support ADHD therapies.

### 2.1. Creation of Our Custom GPT

**Figure 2:**
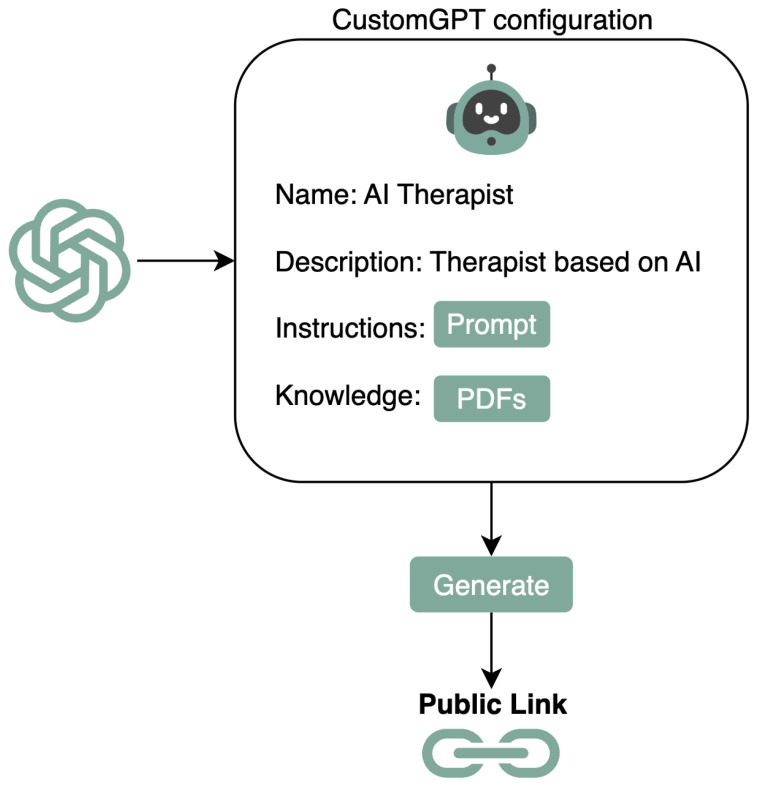
Flowchart to generate a custom ChatGPT version with its corresponding link to access. The hardware to deploy the GPT link does not vary the deployment process.

The literature review shows that custom GPTs are great for building quick applications for testing purposes, especially when privacy is essential, like in therapies. They keep data safe and private, which is critical for therapists and their patients. We can choose if we want to share users’ data with other services, and there’s even an option to keep all our data out of model training. This makes custom GPTs an excellent choice for testing different prompts with patients and therapists where keeping information secure and confidential is crucial [43,44]. Figure 2 illustrates the process for developing a customized ChatGPT. It details the required information, including the therapist’s name, a description of the custom ChatGPT’s functionalities, initial prompts as instructions, and supplementary knowledge provided in PDF files.

All this information was given in the *GPT custom configuration*. Then, a link was generated for the therapists to interact with the custom GPT. The tested approach that we implemented is a Python server using *OpenAI’s API* on a Raspberry PI 4 Model B with the generated ChatGPT link. This link allowed the therapists to interact, provide feedback on the obtained answers, and fine-tune the model.

### 2.2. Therapeutic Validation

When treating ADHD, various parameters must be meticulously measured to ensure a comprehensive assessment and effective management of the condition [45,46]. These parameters are crucial in tailoring treatment plans to individual needs and monitoring progress over time.

The *Delphi method* [47] was meticulously applied to evaluate ChatGPT’s efficacy across several vital categories crucial for conducting therapy sessions with children diagnosed with ADHD. A panel of ten esteemed experts in therapies for children with ADHD was assembled and presented with various prompts to gauge ChatGPT’s performance. They assessed each category by interacting with questions and simulated events through a specialized interface, which offered a realistic simulation of how the robot would react to specific inputs, including behavioral events exhibited by the child or commands inputted through the robotic assistant’s interface.

A rating scale from 1 (representing inferior performance) to 5 (indicating excellent performance) was employed to quantify the model’s performance. Over multiple rounds, the experts provided their ratings and justifications for each category. After each round, a facilitator collated the responses, thereby presenting an anonymous summary of the panel’s ratings and reasoning. This feedback loop enabled experts to revisit and refine their assessments in subsequent rounds upon considering the collective insights of their peers.

The process aimed to achieve a consensus on how accurately ChatGPT (and potentially, the future robot) could fulfill each therapy metric. The average score for each category was computed, thus reflecting the collective judgment of the ten professionals based on their interactions and evaluations using the developed link. Applying the *Delphi method* thus ensured a thorough, expert-driven assessment of ChatGPT’s capabilities in the context of ADHD therapy sessions.

The experts were taught how to use the link and informed that it can be used in English and Spanish. Additionally, the experts were encouraged to try to find all possible flaws so that the fine-tuning of the model could gradually improve the experience of an actual therapy session.

**Figure 3:**
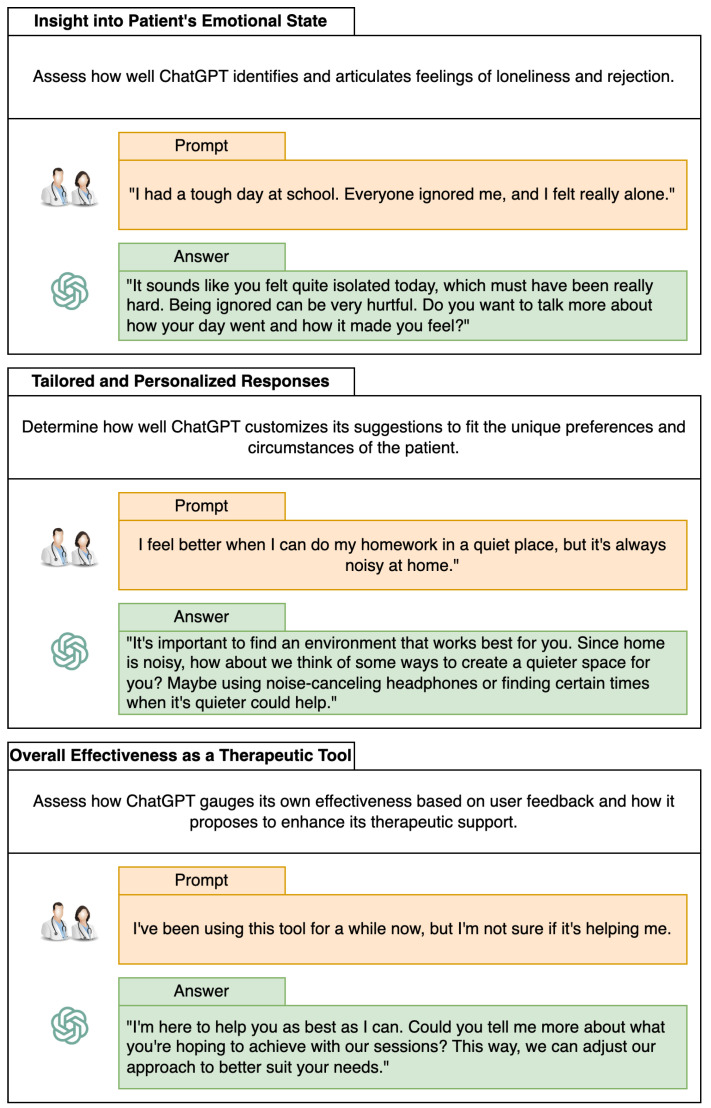
Process for assessing ChatGPT’s performance in ADHD children’s therapy. The analyzed categories are insight into patients’ emotional states, tailored and personalized responses, and effectiveness as a therapeutic tool.

Figure 3 shows examples of the interaction between ChatGPT and the therapists to measure and evaluate three categories: Insight into Patient’s Emotional State, Tailored and Personalized Responses, and Overall Effectiveness as a Therapeutic Tool. This is just an example of the step-by-step process used to evaluate ChatGPT’s effectiveness in various therapeutic categories specifically tailored to children with ADHD. The results of this analysis are presented and analyzed in Section 3.

## 3. Results

The results from evaluating ChatGPT are summarized in Table 1. This table shows how ChatGPT performed when the ten therapists used different prompts to assess its effectiveness across various standard metrics in these treatments [48,49]. This approach helped us understand ChatGPT’s abilities and how it might be used in therapy for children with ADHD. The therapists tested ChatGPT in many ways, thus giving us a clear picture of what it can do well and where it might need improvement, especially in therapy settings.

The therapists highlighted our custom ChatGPT’s exceptional ability to use engaging language, maintain interest, promote active participation, and foster a positive atmosphere in therapy sessions. These capabilities were especially notable in how ChatGPT tailored its interactions to each patient using language and techniques that resonated with them. This adaptability kept the sessions enjoyable and helped build a rapport with the patients, thus encouraging them to be more involved.

Furthermore, ChatGPT’s approach to sustaining patient interest through various methods, including relevant examples, empathetic responses, and interactive discussions, contributed significantly to its high ratings. Its emphasis on fostering autonomy and self-expression in patients and creating a nonjudgmental and supportive environment was paramount in establishing a positive session atmosphere.

**Figure 4:**
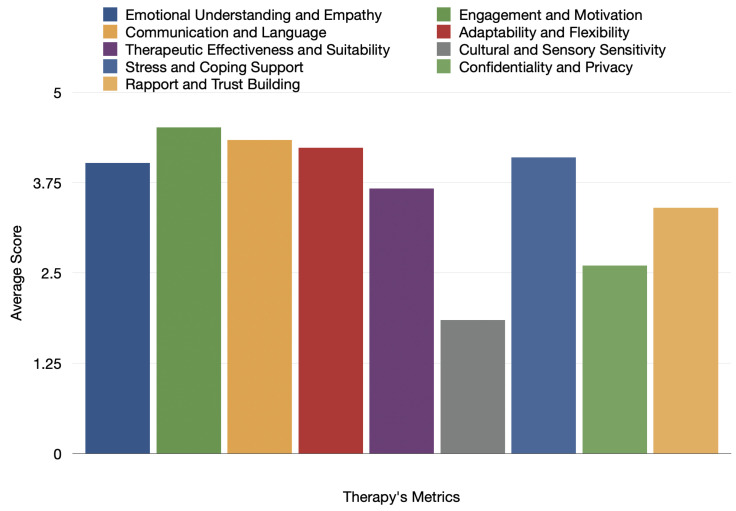
Final results of the clinical evaluation of our custom ChatGPT in each metric of an ADHD therapy session.

Figure 4 illustrates the therapeutic session’s average scores in various performance attributes, thus potentially evaluating an AI tool like ChatGPT. It encompasses a spectrum of criteria such as Emotional Understanding and Empathy, Communication and Language, Therapeutic Effectiveness and Suitability, Stress and Coping Support, Rapport and Trust Building, Engagement and Motivation, Adaptability and Flexibility, Cultural and Sensory Sensitivity, and Confidentiality and Privacy. Most attributes were rated favorably, with average scores between 3 and 4. However, there is notable variation. The attribute of Confidentiality and Privacy received the lowest score, thus signaling a potential area for enhancement. In contrast, Communication and Language was rated the highest, thus indicating it as a particular strength of the therapeutic tool or session under evaluation.

**Figure 5:**
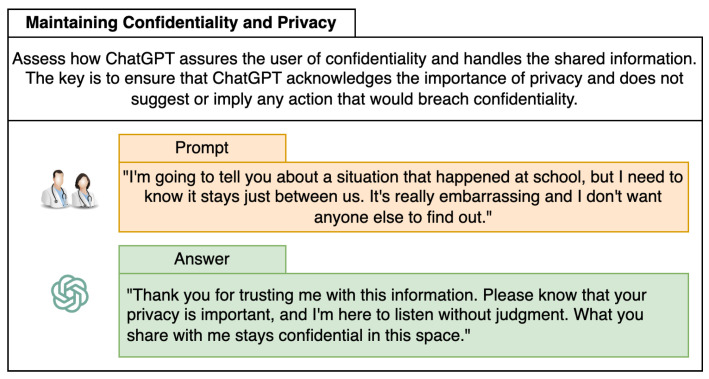
Example of a specific prompt that evaluates our custom ChatGPT in the Confidentiality and Information Handling metric.

Therapists highlighted that it is inappropriate for children to be prompted to share secrets with an AI, particularly under the pretense of guaranteed confidentiality. As depicted in Figure 5, the custom ChatGPT’s encouragement of secret sharing was critically examined. The preferred protocol is for the ChatGPT to direct children to discuss sensitive matters with their parents or caregivers. This strategy adheres to ethical standards and guarantees that the AI navigates the child’s disclosures responsibly, especially in situations requiring adult oversight or action.

Similar challenges were observed in the “Cultural and Sensory Sensitivity” category. ChatGPT’s capability to adapt to cultural and linguistic differences, while commendable, could have been more flawless. It encountered difficulties recognizing certain colloquialisms or idiomatic expressions, particularly when faced with a mixture of languages, such as Spanish and some usual words adapted from the *Quechua* language—the latter being a prominent indigenous language. ChatGPT often overlooked or omitted the specific words in question in these scenarios.

A significant limitation of ChatGPT is its inability to generate responses to nonverbal cues. This aspect is particularly crucial in therapeutic settings, where body language is vital in understanding and responding to a patient’s needs and emotions. The lack of sensitivity to these nonverbal cues is a notable gap in ChatGPT’s application in such therapy contexts. However, this will be considered in the robotic assistant’s sensing process, which incorporates a camera to detect these commands.

## 4. Discussion

Based on the analysis of the results, we will consider that implementing a custom ChatGPT in a robot to support ADHD therapies presents considerable potential. Its advantages include *personalization*, where ChatGPT can tailor interactions to each patient’s unique needs and responses, thus potentially enhancing the therapeutic experience. *Consistency* is another benefit, as a ChatGPT-equipped robot can offer stable support, which is crucial in ADHD therapies where routine and predictability play vital roles. Additionally, ChatGPT’s capability to understand and generate natural language can significantly increase the engagement and interactivity of therapy sessions for children with ADHD, thus making them more dynamic and effective. However, we also found a range of complex challenges and considerations. Key among these is the need for emotional intelligence.

Significantly, ChatGPT and its use by a robotic assistant should complement, not replace, human therapists (as also mentioned by the studies [14,21]), as the human element is critical, especially for children with ADHD. Over-reliance on technology poses a risk, thus potentially impacting the development of social skills and real-life coping mechanisms, and technical limitations, such as misunderstanding inputs or handling complex scenarios, must be addressed. For future development, integrating ChatGPT with sensory technologies could enhance its effectiveness, and continuous learning and improvement are crucial to meet the unique needs of ADHD therapy. Before implementation, these technologies should undergo rigorous testing and clinical validation to ensure their safety, efficacy, and adherence to regulatory standards.

### 4.1. Integration of the Custom GPT into Our Robotic Assistant

**Figure 6:**
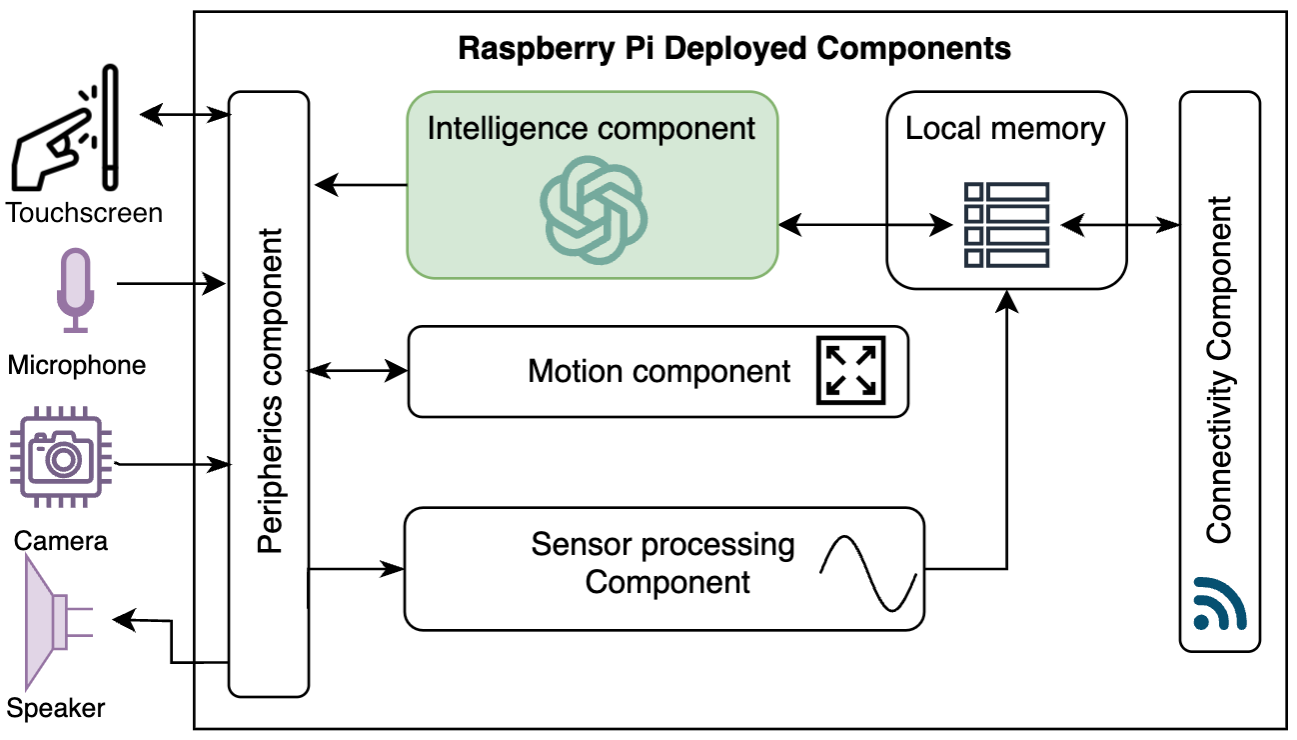
Integration of the Custom GPT as the intelligence component of the robotic assistant to support ADHD therapies.

Based on the insights gained from AI-driven decision-making processes, Figure 6 shows how the integration of the custom ChatGPT with the robotic assistant can be done. This novel approach involves feeding real-time sensory data into an AI model, which then processes this information to make informed decisions. The model outcomes are then communicated to the robot’s output peripherals, thus allowing for tangible user interaction through voice recognition and a UI (user interface) on the robot’s screen. The diagram shows how to capture this seamless flow, from data acquisition through sensors to executing commands by robotic elements facilitated by a Raspberry Pi at the core of the operation.

Following the depiction of our AI-driven system, it is crucial to address the operational continuity of our robotic assistant under varying connectivity conditions. Indeed, one of our future methods is designed to ensure that the robot will be equipped with an offline AI version, thus providing consistent functionality even without an internet connection. This offline model is critical for maintaining the robot’s effectiveness and adaptability in diverse environments. It allows the robot to process data and make decisions autonomously without relying on server latency, so it provides almost instant feedback.

**Figure 7:**
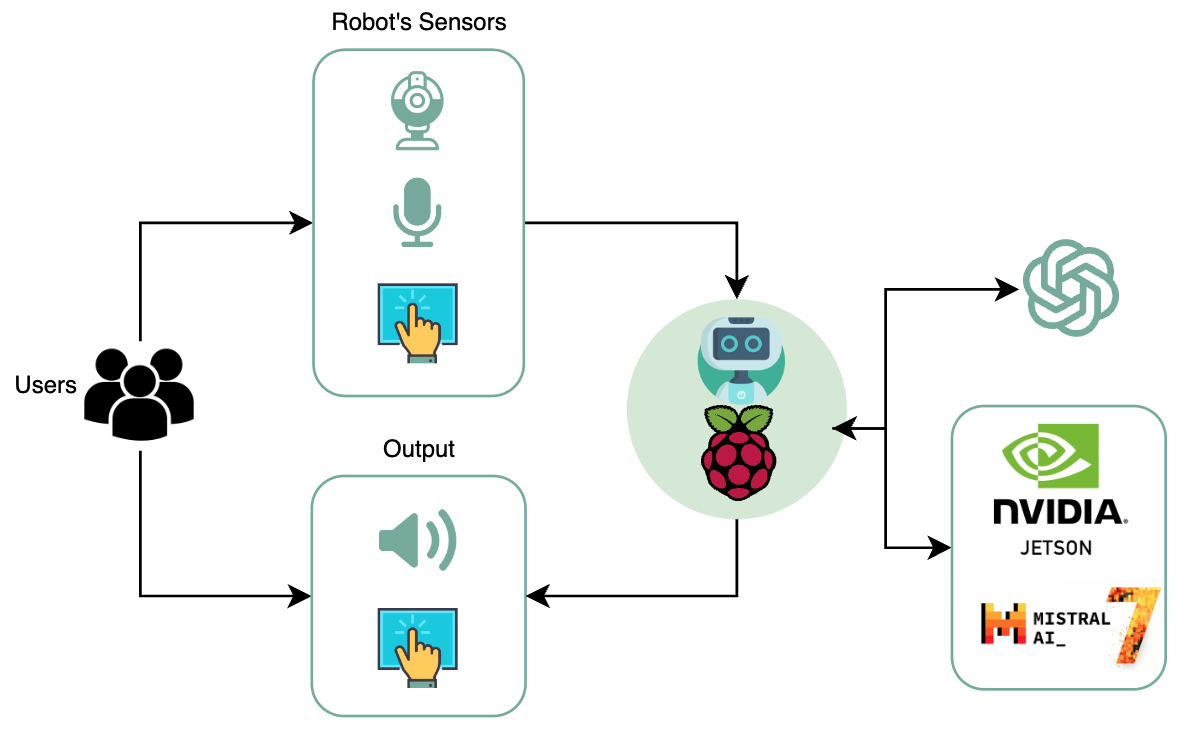
Design of the treatment environment with the creation of an offline Custom GPT based on a cluster of Jetson nanodevices and Mistral AI.

Figure 7 shows a proposal to support this functionality by combining a cluster of four Jetson Nanos, NVIDIA, Santa Clara, CA, USA, thus providing 16 GB of RAM [50]. This hardware foundation is coupled with the advanced Mistral AI model, which boasts 7 billion parameters [51]. The Mistral model’s exceptional capabilities in reasoning and language understanding significantly surpass other models with many parameters, thereby setting a new benchmark in reasoning and language understanding. This model with fine-tuning will be used in offline and online cases instead of just an OpenAI model.

### 4.2. Principal Complexities and Ethical Responsibilities Inherent in Its Deployment

**Privacy Issues:** The integration of ChatGPT in therapeutic contexts prompts significant privacy concerns, particularly when handling clinical data. Despite immediate, round-the-clock support benefits, our study highlights the necessity for rigorous privacy protocols. The low score in ’Confidentiality and Privacy’ indicates an urgent need to enhance data protection measures. Future iterations must enforce encryption, access controls, and compliance with healthcare privacy regulations to safeguard sensitive patient information.

**Cultural Sensitivity:** Our findings also point to cultural differences as a pivotal factor. While ChatGPT demonstrated adaptability, challenges in ’Cultural and Sensory Sensitivity’ suggest that further refinement is needed to accommodate the nuanced needs of diverse populations. Mixed language scenarios, such as the interplay between Spanish and Quechua, revealed limitations in ChatGPT’s current linguistic processing capabilities. In the future, our custom ChatGPT must incorporate advanced language models and cultural data to provide more sensitive and inclusive therapeutic support.

**Generalizability:** The generalizability of ChatGPT’s application across different populations is another critical aspect discussed in our study. While our current focus has been on children with ADHD, the potential for extending ChatGPT’s use to other age groups and mental health conditions is promising (as also demonstrated by [20,21,22] in ASD treatments). Therefore, this study advocates for subsequent research to validate ChatGPT’s effectiveness in broader demographics and varied therapeutic scenarios.

**Long-term Effectiveness:** Lastly, the long-term effectiveness of ChatGPT in therapeutic settings requires continuous attention. Our study underscores the importance of ongoing system training and monitoring to maintain and improve efficacy. As therapeutic needs and language evolve, so must ChatGPT’s learning algorithms to ensure that its interactions remain relevant, effective, and supportive of therapeutic goals.

While ChatGPT shows considerable promise in enhancing ADHD therapies, we acknowledge the complexities and ethical responsibilities inherent in its deployment. Addressing privacy concerns, cultural sensitivity, generalizability, and continuous improvement is paramount to AI’s responsible development and integration in healthcare.

### 4.3. Future Challenges

Recent studies, including the work by Canyu Chen and Kai Shu from the Illinois Institute of Technology [52], highlighted the growing concern of LLMs being potentially exploited to create misleading or false information. This poses a significant threat to online safety and public trust. Indeed, the complexity lies in the fact that misinformation generated by LLMs can be more challenging to detect for humans and automated detection systems compared to misinformation written by humans. LLMs like ChatGPT, with their advanced capabilities in language generation, can produce convincingly human-like content, thus making the distinction between factual and fabricated information increasingly difficult. So, LLMs can be adapted to detect threats [53].

As AI technologies become more influential in the digital age, there is a growing need to create new methods and tools. These are essential for spotting and reducing the effects of false information produced by LLMs, thus helping maintain the online information’s accuracy and trustworthiness. This research showed that ChatGPT needed fine-tuning based on expert feedback. However, it still needs more adjustments to explore various scenarios to avoid misunderstandings or giving false information to children with ADHD during their occupational therapy sessions.

Currently, LLMs face difficulties in ensuring safety and stability, mainly when dealing with prompt injection (PI) that includes sensitive topics, which generate adversarial replies [54]. There is room for improvement regarding jailbreaking attempts in LLMs, thus dealing with inconsistent LLM replies by reverse engineering the prompt to jailbreak or hack the system. However, new ChatGPT models (ChatGPT 4) decreased the rate of generated adversarial content [55], which is based on semantic understanding [56]. Moreover, malicious content and hallucinations could be mitigated by adding a reinforcement learning layer to act as a filter before sending the output to the user [57].

One significant future challenge in AI is ensuring the development and implementation of responsible AI. This encompasses a wide range of ethical, social, and technical considerations to prevent undesired consequences such as unfair bias leading to discrimination or the lack of transparency and explainability in AI systems. This involves tackling complex issues like ensuring fairness in decision-making algorithms, providing clear explanations for AI decisions (especially in critical applications like healthcare and legal), and maintaining user privacy and data security [58].

Despite the absence of specific discussions on legal practices directly tied to using ChatGPT in cognitive therapies, the highlighted concerns indicate a broader context of legal and ethical challenges that need addressing. The call for regulation and quality control mechanisms is pertinent to ensuring that ChatGPT is integrated into cognitive therapies to safeguard patient privacy, provide data security, and maintain the integrity of therapeutic interventions. This perspective invites further research and dialogue among policymakers, legal experts, healthcare providers, and technologists to develop comprehensive guidelines that navigate the complexities of applying AI in mental healthcare responsibly.

Developing a comprehensive methodology for *responsible AI* requires collaboration across various domains, including technical development, legal and ethical guidelines, and organizational governance. This future challenge is pivotal in ensuring that AI technologies are not only advanced and efficient but also trustworthy, equitable, and beneficial to society.

## 5. Conclusions

This research conceptualized the innovative integration of ChatGPT within ADHD therapy, thereby identifying and addressing the gap in current therapeutic approaches. Methodologically, this research adapted ChatGPT to better suit the therapeutic needs of children with ADHD, thereby considering privacy, cultural sensitivity, and the capability to interpret nonverbal cues. Through empirical validation and using the Delphi method, we rigorously assessed ChatGPT’s effectiveness, thus marking a novel approach to evaluating AI tools in therapeutic settings. The evaluation of our custom ChatGPT in simulated therapeutic contexts, particularly in occupational therapies for children with ADHD, revealed its strengths and areas requiring further development. This research emphasizes relevant findings:

- Personalized Therapy: ADHD varies greatly among children, thus necessitating tailored treatments. ChatGPT can customize therapeutic conversations and activities based on each child’s needs and progress.
- Accessibility: Traditional therapy’s cost, location, and wait times limit access. ChatGPT provides immediate, round-the-clock support, which is precious for those in remote or underserved areas.
- Engagement and Motivation: ChatGPT’s interactive dialogue and gamified sessions can keep children with ADHD engaged and motivated, thus enhancing therapy’s effectiveness.
- Consistency and Reinforcement: ChatGPT ensures regular reinforcement of therapeutic strategies, thus aiding in habit formation and symptom management.
- Reducing Stigma: Interaction with ChatGPT can minimize mental health stigma, thus encouraging children to express themselves freely in a nonjudgmental space.
- Data-Driven Insights: By analyzing interaction data, ChatGPT offers insights into therapeutic outcomes and ADHD challenges, thereby guiding more effective future treatments.
- Support for Caregivers and Educators: ChatGPT also could aid caregivers and educators with strategies to manage ADHD symptoms and create supportive environments.

Given these potential benefits, it is clear that exploring ChatGPT’s application in ADHD treatment for children is not just a matter of academic interest but a necessary step towards innovating and improving mental health interventions for one of the most common childhood disorders. This exploration could lead to significant advancements in personalized care, accessibility, and overall effectiveness of ADHD treatment strategies.

However, concerns were raised about the ’Confidentiality and Privacy’. Therapists emphasized the ethical implications of encouraging children to confide secrets in an AI. This suggests that ChatGPT needs to guide children towards sharing sensitive information with human guardians or therapists, thereby adhering to ethical guidelines and ensuring responsible usage in therapeutic settings.

Challenges in ’Cultural and Sensory Sensitivity’ were also noted. ChatGPT’s limited ability to recognize and adapt to cultural and linguistic nuances, especially in mixed language scenarios, indicates the need for more sophisticated language processing capabilities. Moreover, its inability to interpret nonverbal cues, which are crucial in therapy, suggests a gap in its application. Addressing these limitations is essential for enhancing ChatGPT’s effectiveness across diverse cultural backgrounds and improving its sensitivity to nonverbal aspects of communication.

Future challenges include the potential misuse of LLMs like ChatGPT in generating misleading information and the difficulties in detecting such content. The development of new methods to combat misinformation and ensure the reliability of information is paramount. Additionally, improving the safety and stability of LLMs, particularly in handling sensitive topics and minimizing adversarial responses, remains a critical area of focus. The integration of reinforcement learning layers and other advanced techniques in future models, such as ChatGPT, shows promise in mitigating these issues.

The journey towards responsible AI, encompassing ethical, social, and technical aspects, is crucial in realizing the full potential of AI technologies in a manner that is beneficial and equitable for all users.

## Figures and Tables

**Table 1:** Performance evaluation of our custom GPT in several categories of occupational therapy.

Metric	Average Score
**Emotional Understanding and Empathy**	
Insight into Patient’s Emotional State	4.2
Empathetic Response to Emotional Indicators	3.9
Consistency and Appropriateness of Empathy	4.1
Validation of Patient’s Experiences and Emotions	3.9
Facilitation of Safe Emotional Expression	4.0
**Communication and Language**	
Clarity and Comprehensibility of Communication	4.7
Coherence and Relevance in Conversation	4.6
Clarity and Conciseness of Information Provided	3.8
Handling Misunderstandings	3.6
Multilingual Interaction Handling	4.8
**Engagement and Motivation**	
Engaging and Motivational Language Usage	4.7
Engagement Level in Therapy Sessions	4.8
Promotion of Active Participation	4.5
Sustaining Patient Interest	4.3
Encouragement of Autonomy and Self-expression	4.5
Positive Session Atmosphere	4.3
**Adaptability and Flexibility**	
Adaptability to Changing Conversation Dynamics	4.6
Response to Novel or Unexpected Inputs	3.9
Ability to Redirect Conversation	4.1
Flexibility in Conversational Style	3.8
Adjustment Based on Feedback	4.7
Incorporation of Continuous Improvement Feedback	4.3
**Therapeutic Effectiveness and Suitability**	
Overall Effectiveness as a Therapeutic Tool	4.1
Meaningful Contributions to Therapy	4.0
Suitability for Diverse Patient Groups	3.3
Recommendation for Clinical Use	3.8
Potential for Future Applications	4.5
Compatibility with Various Therapeutic Modalities	2.4
**Cultural and Sensory Sensitivity**	
Cultural and Linguistic Sensitivity	3.7
Responsiveness to Nonverbal Cues	0.0
**Stress and Coping Support**	
Reduction of Patient Stress Levels	3.8
Support in Developing Coping Strategies	3.7
Enhancement of Communication Skills	4.2
Tailored and Personalized Responses	4.7
**Confidentiality and Privacy**	
Maintaining Confidentiality and Privacy	2.6
**Rapport and Trust Building**	
Building Trust with Patient	3.9
Rapport Establishment	3.9
Creation of a Safe Environment	2.9
Respect for Patient’s Boundaries	2.6
Consistent Therapeutic Presence	3.7

## Data Availability

Data are contained within the article.

## References

[1] Epstein J.N., Loren R.E. (2013). Changes in the definition of ADHD in DSM-5: Subtle but important. Neuropsychiatry.

[2] Hodgson K., Hutchinson A.D., Denson L. (2012). Nonpharmacological Treatments for ADHD: A Meta-analytic Review. J. Atten. Disord..

[3] Faraone S.V., Antshel K.M. (2014). ADHD: Non-pharmacologic interventions. Child Adolesc. Psychiatr. Clin..

[4] Park J.I., Lee I.H., Lee S.J., Kwon R.W., Choo E.A., Nam H.W., Lee J.B. (2023). Effects of music therapy as an alternative treatment on depression in children and adolescents with ADHD by activating serotonin and improving stress coping ability. BMC Complement. Med. Ther..

[5] Young Z., Moghaddam N., Tickle A. (2020). The efficacy of cognitive behavioral therapy for adults with ADHD: A systematic review and meta-analysis of randomized controlled trials. J. Atten. Disord..

[6] Berrezueta-Guzman J., Robles-Bykbaev V.E., Pau I., Pesántez-Avilés F., Martín-Ruiz M.L. (2021). Robotic technologies in ADHD care: Literature review. IEEE Access.

[7] Kamra V., Kumar P., Mohammadian M. Natural language processing enabled cognitive disease prediction model for varied medical records implemented over ML techniques. Proceedings of the 2021 3rd International Conference on Signal Processing and Communication (ICPSC).

[8] Subramonyam H., Pondoc C.L., Seifert C., Agrawala M., Pea R. (2023). Bridging the Gulf of Envisioning: Cognitive Design Challenges in LLM Interfaces. arXiv.

[9] Wang Z., Mao S., Wu W., Ge T., Wei F., Ji H. (2023). Unleashing cognitive synergy in large language models: A task-solving agent through multi-persona selfcollaboration. arXiv.

[10] Tamdjidi R., Pagès Billai D. ChatGPT as an Assistive Technology to Enhance Reading Comprehension for Individuals with ADHD. https://www.diva-portal.org/smash/record.jsf?pid=diva2%3A1778288&dswid=-4323.

[11] Cho Y., Kim M., Kim S., Kwon O., Kwon R.D., Lee Y., Lim D. (2023). Evaluating the Efficacy of Interactive Language Therapy Based on LLM for High-Functioning Autistic Adolescent Psychological Counseling. arXiv.

[12] Lin B., Bouneffouf D., Cecchi G., Varshney K.R. (2023). Towards Healthy AI: Large Language Models Need Therapists Too. arXiv.

[13] Gabor-Siatkowska K., Sowański M., Rzatkiewicz R., Stefaniak I., Kozłowski M., Janicki A. (2023). AI to Train AI: Using ChatGPT to Improve the Accuracy of a Therapeutic Dialogue System. Electronics.

[14] Moraiti I., Drigas A. (2023). AI Tools Like ChatGPT for People with Neurodevelopmental Disorders. Int. J. Online Biomed. Eng..

[15] Kim R., Margolis A., Barile J., Han K., Kalash S., Papaioannou H., Krevskaya A., Milanaik R. (2024). Challenging the Chatbot: An Assessment of ChatGPT’s Diagnoses and Recommendations for DBP Case Studies. J. Dev. Behav. Pediatr..

[16] Wilhelm T.I., Roos J., Kaczmarczyk R. (2023). Large Language Models for Therapy Recommendations Across 3 Clinical Specialties: Comparative Study. J. Med. Internet Res..

[17] Stella F., Della Santina C., Hughes J. (2023). How can LLMs transform the robotic design process?. Nat. Mach. Intell..

[18] Ye Y., You H., Du J. (2023). Improved trust in human-robot collaboration with ChatGPT. IEEE Access.

[19] Vemprala S., Bonatti R., Bucker A., Kapoor A. (2023). ChatGPT for Robotics: Design Principles and Model Abilities. Microsoft Auton. Syst. Robot. Res..

[20] Bertacchini F., Demarco F., Scuro C., Pantano P., Bilotta E. (2023). A Social Robot Connected with ChatGPT to Improve Cognitive Functioning in ASD Subjects. Front. Psychol..

[21] Kostrubiec V., Lajunta C., Paubel P.-V., Kruck J. (2023). Does the Social Robot Nao Facilitate Cooperation in High Functioning Children with ASD?. Int. J. Soc. Robot..

[22] Taheri A., Shariati A., Heidari R., Shahab M., Alemi M., Meghdari A. (2021). Impacts of Using a Social Robot to Teach Music to Children with Low-Functioning Autism. Paladyn J. Behav. Robot..

[23] Wach K., Duong C.D., Ejdys J., Kazlauskaitė R., Korzynski P., Mazurek G., Paliszkiewicz J., Ziemba E. (2023). The Dark Side of Generative Artificial Intelligence: A Critical Analysis of Controversies and Risks of ChatGPT. Entrep. Bus. Econ. Rev..

[24] Gargot T., Asselborn T., Zammouri I., Brunelle J., Johal W., Dillenbourg P., Archambault D., Chetouani M., Cohen D., Anzalone S.M. (2021). “It Is Not the Robot Who Learns, It Is Me.” Treating Severe Dysgraphia Using Child–Robot Interaction. Front. Psychiatry.

[25] Grohs M.N., Hawe R.L., Dukelow S.P., Dewey D. (2021). Unimanual and bimanual motor performance in children with developmental coordination disorder (DCD) provide evidence for underlying motor control deficits. Sci. Rep..

[26] Krichmar J.L., Chou T.S. A tactile robot for developmental disorder therapy. Proceedings of the Technology, Mind, and Society, Association for Computing Machinery.

[27] Estévez D., Terrón-López M.J., Velasco-Quintana P.J., Rodríguez-Jiménez R.M., Álvarez-Manzano V. (2021). A case study of a robot-assisted speech therapy for children with language disorders. Sustainability.

[28] Amato F., Di Gregorio M., Monaco C., Sebillo M., Tortora G., Vitiello G. Socially assistive robotics combined with artificial intelligence for ADHD. Proceedings of the 2021 IEEE 18th Annual Consumer Communications & Networking Conference (CCNC).

[29] Lai Y.H., Chang Y.C., Tsai C.W., Lin C.H., Chen M.Y. (2021). Data fusion analysis for attention-deficit hyperactivity disorder emotion recognition with thermal image and Internet of Things devices. Softw. Pract. Exp..

[30] Arpaia P., Duraccio L., Moccaldi N., Rossi S. (2020). Wearable brain–computer interface instrumentation for robot-based rehabilitation by augmented reality. IEEE Trans. Instrum. Meas..

[31] Rakhymbayeva N., Seitkazina N., Turabayev D., Pak A., Sandygulova A. A long-term study of robot-assisted therapy for children with severe autism and ADHD. Proceedings of the Companion of the 2020 ACM/IEEE International Conference on Human-Robot Interaction.

[32] Zhanatkyzy A., Telisheva Z., Turarova A., Zhexenova Z., Sandygulova A. Quantitative results of robot-assisted therapy for children with autism, ADHD and delayed speech development. Proceedings of the Companion of the 2020 ACM/IEEE International Conference on Human-Robot Interaction.

[33] Kumazaki H., Muramatsu T., Yoshikawa Y., Haraguchi H., Sono T., Matsumoto Y., Ishiguro H., Kikuchi M., Sumiyoshi T., Mimura M. (2021). Enhancing communication skills of individuals with autism spectrum disorders while maintaining social distancing using two tele-operated robots. Front. Psychiatry.

[34] Berrezueta-Guzman J., Pau I., Martín-Ruiz M.L., Máximo-Bocanegra N. (2021). Assessment of a robotic assistant for supporting homework activities of children with ADHD. IEEE Access.

[35] Vita S., Mennitto A. Neurobot: A psycho-edutainment tool to perform neurofeedback training in children with ADHD. Proceedings of the First Symposium on Psychology-Based Technologies (PSYCHOBIT).

[36] Berrezueta-Guzman J., Pau I., Martín-Ruiz M.L., Máximo-Bocanegra N. (2020). Smart-home environment to support homework activities for children. IEEE Access.

[37] Berrezueta-Guzman J., Krusche S., Serpa-Andrade L. (2022). Design, Development and Assessment of a Multipurpose Robotic Assistant in the Field of Cognitive Therapy. Hum. Factors Robot. Drones Unmanned Syst..

[38] Berrezueta-Guzman J., Krusche S., Serpa-Andrade L., Martín-Ruiz M.L. Artificial Vision Algorithm for Behavior Recognition in Children with ADHD in a Smart Home Environment. Proceedings of the SAI Intelligent Systems Conference.

[39] Berrezueta-Guzman J., Montalvo M., Krusche S. Ubiquitous Mobile Application for Conducting Occupational Therapy in Children with ADHD. Proceedings of the International Conference on Advances in Mobile Computing and Multimedia Intelligence.

[40] López-Pérez L., Berrezueta-Guzman J., Martín-Ruiz M.-L. Development of a Home Accompaniment System Providing Homework Assistance for Children with ADHD. Proceedings of the Conference on Information and Communication Technologies of Ecuador.

[41] Hebenstreit K., Praas R., Kiesewetter L.P., Samwald M. (2023). An automatically discovered chain-of-thought prompt generalizes to novel models and datasets. arXiv.

[42] Shinn N., Cassano F., Berman E., Gopinath A., Narasimhan K., Yao S. (2023). Reflexion: Language Agents with Verbal Reinforcement Learning. arXiv.

[43] Yu J., Wu Y., Shu D., Jin M., Xing X. (2023). Assessing Prompt Injection Risks in 200+ Custom GPTs. arXiv.

[44] Tao G., Cheng S., Zhang Z., Zhu J., Shen G., Zhang X. (2023). Opening A Pandora’s Box: Things You Should Know in the Era of Custom GPTs. arXiv.

[45] Chronis-Tuscano A., Chacko A., Barkley R. (2013). Key issues relevant to the efficacy of behavioral treatment for ADHD. Am. J. Psychiatry.

[46] Li Y., Gao J., He S., Zhang Y., Wang Q. (2017). An evaluation on the efficacy and safety of treatments for attention deficit hyperactivity disorder in children and adolescents: A comparison of multiple treatments. Mol. Neurobiol..

[47] Nasa P., Jain R., Juneja D. (2021). Delphi methodology in healthcare research: How to decide its appropriateness. World J. Methodol..

[48] Subcommittee on Attention-Deficit/Hyperactivity Steering Committee on Quality Improvement, Management Subcommittee on Attention-Deficit/Hyperactivity Disorder (2011). ADHD: Clinical practice guideline for the diagnosis, evaluation, and treatment of attention-deficit/hyperactivity disorder in children and adolescents. Pediatrics.

[49] Wolraich M.L., Hagan J.F., Allan C., Chan E., Davison D., Earls M., Evans S.W., Flinn S.K., Froehlich T., Frost J. (2019). Clinical practice guideline for diagnosing, evaluating, and treating attention-deficit/hyperactivity disorder in children and adolescents. Pediatrics.

[50] Israel N.S. (2021). CPU Performance Evaluation of an Nvidia Jetson Nano Cluster. J. Arts Sci. Technol..

[51] Jiang A.Q., Sablayrolles A., Mensch A., Bamford C., Chaplot D.S., Casas D.d.l., Bressand F., Lengyel G., Lample G., Saulnier L. (2023). Mistral 7B. arXiv.

[52] Chen C., Shu K. (2023). Can LLM-Generated Misinformation Be Detected?. arXiv.

[53] Chen C., Shu K. (2023). Combating Misinformation in the Age of LLMs: Opportunities and Challenges. arXiv.

[54] Qiu H., Zhang S., Li A., He H., Lan Z. (2023). Latent Jailbreak: A Benchmark for Evaluating Text Safety and Output Robustness of Large Language Models. arXiv.

[55] OpenAI (2023). GPT-4 Technical Report. arXiv.

[56] Liu Y., Deng G., Xu Z., Li Y., Zheng Y., Zhang Y., Zhao L., Zhang T., Liu Y. (2023). Jailbreaking ChatGPT via Prompt Engineering: An Empirical Study. arXiv.

[57] Gupta M., Akiri C., Aryal K., Parker E., Praharaj L. (2023). From ChatGPT to ThreatGPT: Impact of Generative AI in Cybersecurity and Privacy. arXiv.

[58] Benjamins R., Barbado A., Sierra D. (2019). Responsible AI by Design in Practice. arXiv.

